# Non‐interventional, prospective, observational study on spasticity‐associated symptom control with nabiximols as add‐on therapy in patients with multiple sclerosis spasticity in Austria

**DOI:** 10.1002/brb3.2947

**Published:** 2023-03-19

**Authors:** Michael Guger, Robert Hatschenberger, Fritz Leutmezer

**Affiliations:** ^1^ Department of Neurology Pyhrn‐Eisenwurzen Klinikum Steyr Steyr Austria; ^2^ Department of Neurology Klinikum Bad Hall and Bad Schallerbach Bad Schallerbach Austria; ^3^ Department of Neurology Medical University Vienna Vienna Austria

**Keywords:** multiple sclerosis, nabiximols, observational study, spasticity‐associated symptoms

## Abstract

**Background and purpose:**

Randomized controlled trials and observational studies of nabiximols oromucosal spray in patients with multiple sclerosis (MS) spasticity have shown improvement in a range of associated symptoms (pain, spasms, fatigue, bladder dysfunction, and sleep disturbances). This study evaluated the effectiveness and tolerability of add‐on nabiximols in the routine management of patients with MS spasticity in Austria, with a focus on spasticity‐associated symptoms.

**Methods:**

This was an open, prospective, multicenter, observational, non‐interventional study of patients with MS spasticity receiving add‐on treatment with nabiximols oromucosal spray. Main endpoints were patient‐reported changes from baseline in the frequency (counts) or severity (mean Numerical Rating Scale [NRS] scores) of spasticity‐associated symptoms, and patient‐reported changes from baseline in impairment of daily activities due to spasticity, after 1 and 3 months of nabiximols treatment. No analyses were conducted for statistical significance.

**Results:**

There were 55 patients in the effectiveness population, and 62 in the safety population. Patients reported clinically relevant reductions from baseline to month 3 in the average number of spasms/day (−68.2%) and number of urinary incontinence episodes (−69.3%) in the week prior to the clinic visit, and reductions in mean 0−10 NRS scores for sleep impairment (−47.2%), fatigue (−26.4%), pain (40.4%), and spasticity severity (39.0%). There was no change from baseline in daily activity impairment due to spasticity. The majority of patients were at least partly satisfied with add‐on nabiximols for spasticity‐associated symptoms. There were 31 adverse events (27 treatment related) reported in 19 patients, with no new safety signals.

**Conclusions:**

Add‐on nabiximols improved the severity of MS spasticity and a range of spasticity‐associated symptoms during real‐world use in Austria. Nabiximols is an option for patients with MS spasticity who fail first‐line oral antispasticity treatment.

## Graphical abstract

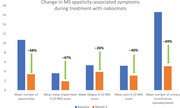

## 1 INTRODUCTION

Spasticity, characterized by a velocity‐dependent increase in muscle tone, is a common symptom of multiple sclerosis (MS), occurring with at least moderate severity in about 40% of patients with MS as the disease evolves (Kister et al., 2013; Oreja‐Guevara et al., 2013). Spasticity can further restrict mobility and is associated with symptoms such as pain, spasms, fatigue, bladder dysfunction, and sleep disturbances (Flachenecker et al., 2014a; Kister et al., 2013), with a cumulative negative impact on patients’ daily activities and quality of life (Arroyo et al., 2013; Flachenecker et al., 2014a; Zwibel, 2009). Early and effective treatment of MS spasticity is essential to minimize patient burden and avoid longer term sequalae.

Spasticity treatment consists mainly of physiotherapy and pharmacotherapy (Flachenecker et al., 2014a; Otero‐Romero et al., 2016); however, conventional oral antispasticity agents have limited efficacy (Paisley et al., 2002) and treatment failure is common. Nabiximols oromucosal spray (Sativex^®^; GW Pharmaceuticals, Cambridge, UK) is a complex botanical mixture containing delta‐9‐tetrahydrocannibinol (THC), cannabidiol (CBD), and other plant‐derived cannabinoids and non‐cannabinoid constituents (Russo & Guy, 2006). Extensive experience with nabiximols in clinical trials and everyday practice indicates that a relevant proportion of patients can derive meaningful and durable symptomatic relief of MS spasticity (Chan & Silván, 2022; Conte & Vila Silván, 2021), which can improve balance and walking (De Blasiis et al., 2021). Nabiximols is approved as an add‐on treatment to oral antispasticity agents for symptomatic management of MS spasticity (eMC, Summary of Product Characteristics, 2022).

The improvement in spasticity‐associated symptoms (e.g. spasms, pain, sleep disturbances) observed with nabiximols in randomized controlled trials and observational studies (Flachenecker et al., 2014b; Markovà et al., 2019; Novotna et al., 2011; Vermersch & Trojano, 2016; Wade et al., 2004) suggested a need to look beyond spasticity itself when assessing clinical benefit in patients with MS. Retrospective analyses of real‐world data have since shown that spasticity‐associated symptoms may ameliorate during nabiximols treatment irrespective of the extent of improvement in a patient's spasticity (Patti et al., 2022, 2020).

The objective of this observational study was to evaluate the effectiveness and tolerability of add‐on nabiximols in the routine management of patients with MS spasticity in Austria, with a focus on its effect on spasticity‐associated symptoms.

## 2 METHODS

This was an open, prospective, multicenter, observational, non‐interventional study of patients with MS spasticity receiving add‐on treatment with nabiximols oromucosal spray in accordance with its approved label (eMC, Summary of Product Characteristics, 2022) at MS centers across Austria. Patients were selected according to standard routine assessments and study inclusion took place after a therapeutic decision had been taken to start nabiximols treatment. Exclusion criteria were hypersensitivity to cannabinoids or any excipients, any known or suspected history or family history of schizophrenia or other psychotic disease, history of severe personality disorder or other significant psychiatric disorder other than depression associated with the underlying condition, and breast feeding (eMC, Summary of Product Characteristics, 2022).

The study was approved by the Ethics Commission of the Medical Faculty of Johannes Kepler University Linz. Study planning, execution, and evaluation were based on scientific guidelines for implementing non‐interventional studies in Austria (Bundesamt für Sicherheit im Gesundheitswesen, 2018). The study was conducted in accordance with relevant legal obligations for data processing and data protection (General Data Protection Regulation (EU) 2016/679). All data were deidentified. All patients provided written informed consent prior to study participation.

### 2.1 Data collection

Data were collected prospectively over a 3‐month period: at baseline and at 1 month and 3 months after the start of nabiximols treatment. In patients who discontinued nabiximols prior to the last scheduled visit, data were collected at an early termination visit.

Validated instruments were used to measure symptom severity. Case report forms and patient questionnaires were used to collect patient data. The severity of spasticity and of sleep disturbances, fatigue, and pain due to spasticity‐associated symptoms was assessed using the corresponding patient‐rated 0−10 Numerical Rating Scale (NRS), where 0 = none to 10 = worst imaginable. A ≥20% reduction from baseline in symptom severity on the 0−10 NRS represents a minimal clinically important difference and a ≥30% reduction represents a clinically important difference (Farrar et al., 2008). The average number of spasms/day during the week before the clinic visit, the number of awakenings/night due to spasticity‐associated symptoms, and the number of urinary incontinence episodes during the week before the clinic visit were assessed by counts.

At baseline, investigators collected data on patient demographics, MS history, the presence and frequency/severity of spasticity‐associated symptoms, spasticity severity, and previous and current concomitant therapy for spasticity‐associated symptoms. At 1 and 3 months after nabiximols start, data were recorded on the presence and frequency/severity of spasticity‐associated symptoms, spasticity severity, current concomitant therapy for spasticity‐associated symptoms, nabiximols dose/regimen, occurrence of adverse events (AEs), and pregnancies or special situations. For patients who terminated nabiximols prior to 3 months’ observation, additional items recorded at the early termination visit were date and reason for discontinuation.

Patients were given a questionnaire at baseline and after 1 and 3 months of nabiximols treatment to record their assessment of spasticity‐associated symptoms, which included spasticity severity (NRS); localization of spasticity‐associated symptoms; frequency of spasticity‐associated symptoms per day; severity of sleep disturbances (NRS), fatigue (NRS), and pain (NRS); frequency of urinary incontinence episodes/week; impairment of daily activities due to spasticity (five‐point categorical scale ranging from “Spasticity does not restrict me in my activities” to “Every day spasticity prevents me from being able to perform many of my day‐to‐day activities”); and most troublesome symptoms. Patients completed a separate questionnaire to record their satisfaction (at baseline) with previous therapy for spasticity‐associated symptoms, and their satisfaction (at 1 and 3 months) with add‐on nabiximols for spasticity‐associated symptoms. Treatment satisfaction was assessed on a 6‐point categorical scale, ranging from “very dissatisfied” to “very satisfied.”

### 2.2 Outcomes

The main endpoints were patient‐reported changes from baseline in the frequency (counts) or severity (mean 0−10 NRS scores) of spasticity‐associated symptoms, and patient‐reported changes from baseline in impairment of daily activities due to spasticity, after 1 and 3 months of nabiximols treatment. Secondary endpoints were patient‐reported changes from baseline in NRS spasticity severity and patients’ satisfaction with add‐on nabiximols therapy, after 1 and 3 months of nabiximols treatment.

### 2.3 Tolerability

In accordance with pharmacovigilance regulations, AEs were monitored continuously throughout the 3‐month observation period. Information collected about AEs (whether observed by the investigator or reported by the subject) included date of occurrence, relationship to study medication, assessment of intensity, action taken with respect to study medication, event outcome, and judgment as to whether the AEs were “serious” or “non‐serious”. AEs were coded according to MedDRA primary system organ class and preferred term.

### 2.4 Statistical analyses

Statistical analysis was performed using Statistical Analysis System (SAS) version 9.4 (SAS/STAT 14.3) for Windows (SAS Institute, Cary, NC, USA). Descriptive statistics were used. Continuous variables are summarized as mean and standard deviation (mean ± SD). Categorical variables are summarized as number counts (n) and frequencies (%). No analyses were conducted for statistical significance.

## 3 RESULTS

### 3.1 Patient disposition

**FIGURE 1:**
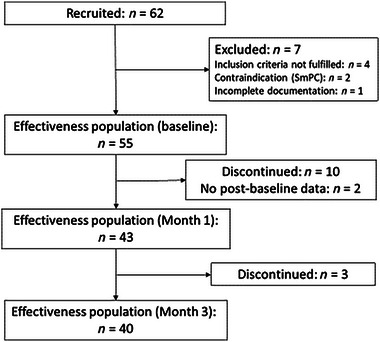
Patient disposition.

A total of 62 patients were recruited from 18 centers in Austria. Patient disposition is shown in Figure 1. Seven patients were excluded from the effectiveness analysis because inclusion criteria were not met (*n* = 4), contraindications (*n* = 2), or incomplete documentation (*n* = 1). Of 55 patients in the effectiveness analysis set, 13 (23.6%) discontinued treatment with nabiximols prior to 3 months’ observation, most during the titration phase, and due to insufficient tolerability, insufficient efficacy, or other reason. In two patients, data were available for baseline visits only. Forty (72.7%) patients completed 3 months’ observation as planned. Data for all 62 patients were analyzed for safety.

The observation period was 3 June 2020 to 10 November 2021. The mean length of observation per patient was 89.0 ± 49.1 days.

The mean nabiximols dose was 7.3 ± 3.7 sprays/day at 1 month, and 7.2 ± 3.3 sprays/day at 3 months.

### 3.2 Patient demographics and clinical characteristics

Patient demographics and clinical characteristics at baseline are summarized in Table 1. The population was 60% female, and the mean age was 52.5 ± 9.6 years. Mean disease duration was 15.1 ± 9.1 years, and approximately half of the cohort (50.9%) had secondary progressive MS. The mean Expanded Disability Status Scale (EDSS) score was 5.3 ± 1.3 points (median 6.0 [range 2.0−7.5]), and the mean spasticity 0−10 NRS score was 6.3 ± 1.6 points. About half of the group (52.7%) had concomitant morbidities, most commonly hypertension and/or MS‐associated depression. Spasticity‐associated symptoms had been present for a mean of 7.7 ± 5.4 years before the baseline visit.

**TABLE 1:** Baseline demographic and clinical characteristics of the effectiveness population (*n* = 55)

Characteristic	Value
Female gender, *n* (%)	33 (60)
Age, years, mean ± SD	52.5 ± 9.6
Duration since MS diagnosis, years, mean ± SD	15.1 ± 9.1
MS type, *n* (%)	
Primary progressive MS	18 (32.7)
Secondary progressive MS	28 (50.9)
Relapsing‐remitting MS	9 (16.4)
0−10 EDSS, mean ± SD	5.3 ± 1.3
0−10 NRS, mean ± SD	6.3 ± 1.6
Concomitant comorbidities^a^, *n* (%)	29 (52.7)
Hypertension^b^	9 (16.4)
Depression^b^	8 (14.5)
Restless legs syndrome^b^	4 (7.3)
Duration of spasticity‐associated symptoms, years, mean ± SD	7.7 ± 5.4
Spasticity‐associated symptoms^a^, *n* (%)	
Increased muscle tone/stiffness	51 (92.7)
Spontaneous muscular activity/seizures	45 (81.8)
Restricted mobility	52 (94.5)
Pain	44 (80.0)
Fatigue	39 (70.9)
Sleep disturbances	30 (54.5)
Bladder dysfunction	38 (69.1)
Concomitant non‐pharmaceutical therapy, *n* (%)	34 (61.8)
Physiotherapy	31 (56.4)
Concomitant antispasticity treatment, *n* (%)	35 (63.6)
Baclofen	16 (29.0)
Tizanidine	13 (23.6)
Fampridine	5 (9.1)
Pregabalin	4 (7.3)
Other^c^	7 (12.7)

Abbreviations: EDSS, Expanded Disability Status Scale; MS, multiple sclerosis; NRS, numerical rating scale.

^a^ Multiple answers were possible.

^b^ Most common concomitant comorbidities (present in ≥4 patients).

^c^ Botulinum toxin *n* = 1, carbamazepine *n* = 2, gabapentin *n* = 1, pramipexole *n* = 2, and tetrahydrocannabinol *n* = 1.

The most common spasticity‐associated symptoms were restricted mobility (94.5%) and increased muscle tone/stiffness (92.7%), mostly of moderate or severe intensity. Previous non‐pharmaceutical therapy of spasticity‐associated symptoms was documented in 36 patients (65.5%) and consisted mainly of physiotherapy (32/36, 88.9%). Previous pharmacotherapy of spasticity‐associated symptoms was documented in 52 patients (94.5%), and consisted mainly of baclofen (39/52, 75.0%) and tizanidine (32/52, 61.5%). At the time of starting nabiximols, 34 patients (61.8%) were receiving a concomitant non‐pharmaceutical therapy, mainly physiotherapy (31/34, 91.2%), and 35 patients (63.6%) were receiving concomitant antispasticity drug therapies, most commonly baclofen (16/35, 45.7%) and/or tizanidine (13/35, 37.1%).

Main reasons for patients to start add‐on nabiximols were insufficient effectiveness of (87.3%) and/or intolerance to (25.5%) previous therapies.

### 3.3 Patient‐reported effectiveness outcomes

#### 3.3.1 Main endpoints

Spasticity‐associated symptoms were localized in the legs in most patients (92.7%). Patient‐assessed frequency/severity of spasticity‐associated symptoms at baseline and after 1 and 3 months of nabiximols treatment is summarized in Table 2.

**TABLE 2:** Patients’ assessment of spasticity‐associated symptoms (via questionnaires) at baseline and during study treatment

Symptom	Baseline (*n* = 55)	Month 1 (*n* = 43)	Month 3 (*n* = 40)
Number of patients with spasms during the week before clinic visit, *n* (%)	37 (67.3)	31 (72.1)	27 (67.5)
Average number of spasms/day during the week before clinic visit, mean ± SD	10.7 ± 12.7	9.6 ± 19.0	3.4 ± 2.6
Sleep impairment, 0−10 NRS, mean ± SD	3.6 ± 2.7	2.0 ± 2.1	1.9 ± 1.8
Number of patients with night awakenings, *n* (%)	33 (60.0)	15 (34.9)	15 (37.5)
Number of night awakenings, mean ± SD	2.3 ± 1.1	3.3 ± 3.5	2.4 ± 2.0
Fatigue, 0−10 NRS, mean ± SD	5.3 ± 2.5	4.5 ± 2.4	3.9 ± 2.3^a^
Pain, 0−10 NRS, mean ± SD	5.2 ± 2.8	4.0 ± 2.6	3.1 ± 2.6
Number of patients with urinary incontinence during the week before clinic visit, *n* (%)	22 (40.0)	17 (39.5)	17 (42.5)
Number of episodes of urinary incontinence during the week before clinic visit, mean ± SD	16.6 ± 24.1	8.8 ± 13.9	5.1 ± 4.5
Spasticity severity within previous 24 h before clinic visit, 0−10 NRS, mean ± SD	6.4 ± 1.8	4.8 ± 1.7	3.9 ± 2.0^b^

Abbreviations: MS, multiple sclerosis; NRS, numerical rating scale.

^a^ *n* = 39

^b^ *n* = 38

At baseline, 67.3% of patients reported experiencing spasms; the proportion was similar at 1 month (72.1%) and 3 months (67.5%). At baseline, patients with spasms reported a mean of 10.7 ± 12.7 spasms/day. The mean number of spasms/day decreased to 9.6 ± 19.0 at 1 month and 3.4 ± 2.6 at 3 months, representing reductions from baseline of 10.3% and 68.2%, respectively.

At baseline, the mean sleep impairment NRS score was 3.6 ± 2.7 points. The mean score decreased to 2.0 ± 2.1 points at 1 month and to 1.9 ± 1.8 points at 3 months, representing reductions from baseline of 44.4% and 47.2%, respectively. At baseline, 60% of patients reported night awakenings due to spasticity symptoms, decreasing to 34.9% of patients at 1 month and 37.5% of patients at 3 months. The mean number of awakenings/night reported by affected patients at each time point was 2.3 ± 1.1 at baseline, 3.3 ± 3.5 at 1 month and 2.4 ± 2.0 at 3 months, equating to increases versus baseline of 43.4% and 4.3%, respectively.

The mean baseline NRS score for fatigue due to spasticity‐associated symptoms was 5.3 ± 2.5 points. The mean fatigue NRS score decreased to 4.5 ± 2.4 points after 1 month and to 3.9 ± 2.3 points after 3 months of nabiximols treatment, representing mean improvement from baseline of 15.1% and 26.4%, respectively.

The mean baseline NRS score for pain due to spasticity‐associated symptoms was 5.2 ± 2.8 points. The reduction in mean pain NRS scores to 4.0 ± 2.6 points after 1 month and 3.1 ± 2.6 points after 3 months of nabiximols treatment was equivalent to mean improvement from baseline of 23.1% and 40.4%, respectively.

At baseline, 22 patients (40.0%) reported the presence of urinary incontinence during the week before the clinic visit. Proportions were similar at 1 month (39.5%) and 3 months (42.5%). Patients with urinary incontinence reported a mean of 16.6 ± 24.1 episodes/week at baseline, 8.8 ± 13.9 episodes/week after 1 month and 5.1 ± 4.5 episodes/week after 3 months of nabiximols treatment, corresponding to improvement from baseline of 47.0% and 69.3%, respectively.

**FIGURE 2:**
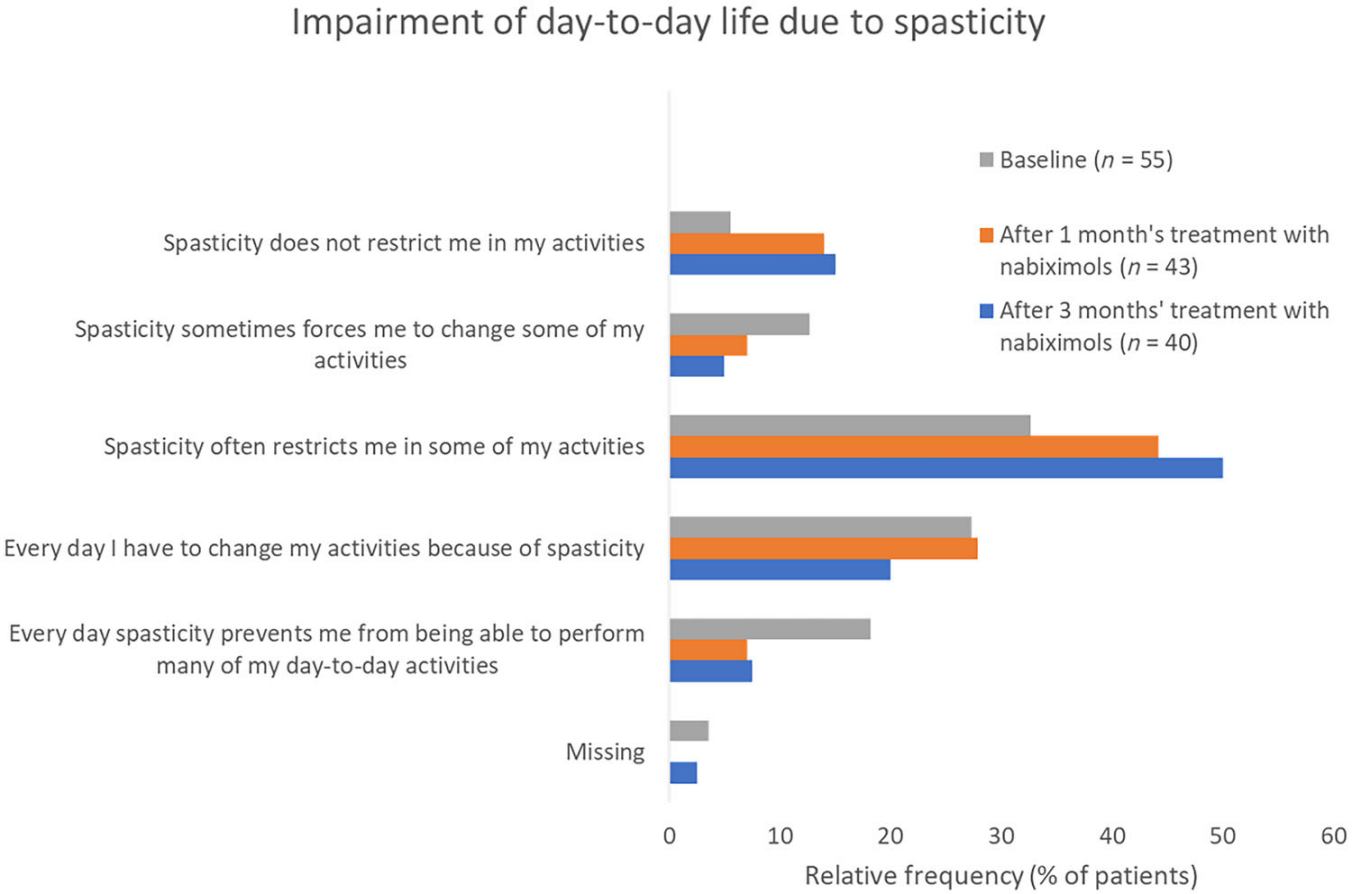
Spasticity‐related impairment of patients’ day‐to‐day life at baseline and after 1 and 3 months of add‐on nabiximols treatment.

At baseline, 78.2% of patients with evaluable data (*n* = 55) reported that spasticity impaired their day‐to‐day life activities at least “often” (several times per week) or daily. This proportion remained consistent throughout 3 months’ observation. The proportion of patients reporting at least frequent impairment of day‐to‐day activities due to spasticity was 79.1% after 1 month (*n* = 43) and 75.5% after 3 months (*n* = 40) of nabiximols treatment (Figure 2).

At baseline, patients’ most disturbing symptoms were muscle stiffness (74.5%) and restricted mobility (80%); this did not change during study treatment. The most frequent troublesome symptoms were muscle stiffness (81.4%) and restricted mobility (67.4%) at 1 month and muscle stiffness (80.0%) and restricted mobility (60.0%) at 3 months.

#### 3.3.2 Secondary endpoints

The mean 0−10 NRS score at baseline for spasticity severity over the previous 24 hours was 6.4 ± 1.8 points. Mean spasticity NRS scores were 4.8 ± 1.7 points after 1 month and 3.9 ± 2.0 points after 3 months, corresponding to improvement from baseline of 25% and 39.0%, respectively.

**FIGURE 3:**
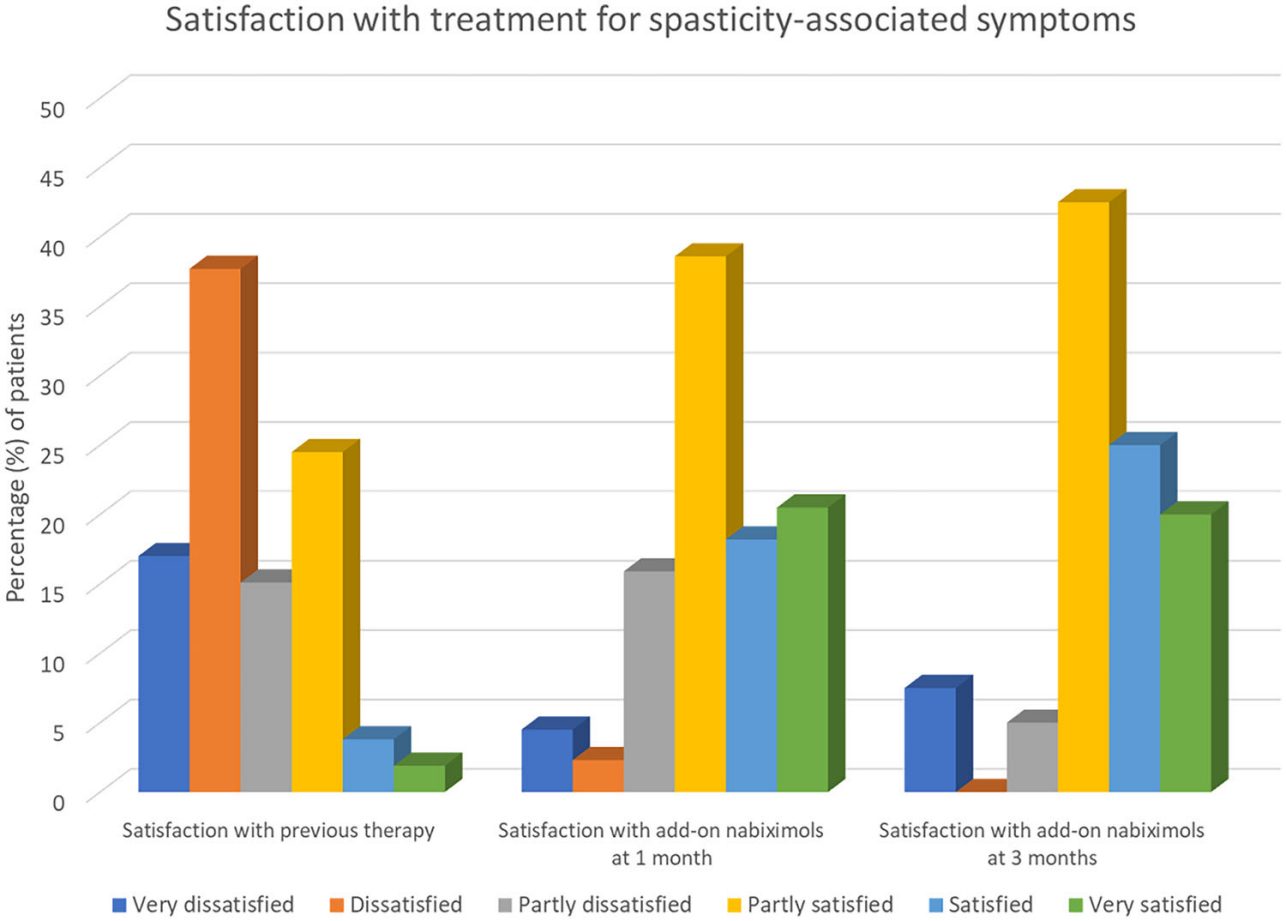
Satisfaction with previous therapy for spasticity‐associated symptoms and satisfaction with add‐on nabiximols after 1 and 3 months of treatment.

At baseline, 30.2% of patients reported being partly satisfied, satisfied, or very satisfied with their previous therapy for spasticity‐associated symptoms. During the study, the proportion of patients who were partly satisfied, satisfied, or very satisfied with add‐on nabiximols for spasticity‐associated symptoms was 77.3% after 1 month and 87.5% after 3 months (Figure 3).

### 3.4 Safety outcomes

A total of 31 AEs were recorded in 19 patients (30.6% of the safety population), of which 27 (87.1%) were considered to be related to nabiximols treatment (i.e., adverse drug reactions). The most common AEs were fatigue (*n* = 5 events), vertigo (*n* = 5), asthenia (*n* = 4), pain (*n* = 2), and somnolence (*n* = 2); all other AEs were reported once only (Table 3). AE intensity was reported as mild (25.8%), moderate (38.7%), or severe (35.5%). Approximately two‐thirds (64.5%) of AEs had recovered or improved at the time of documentation. One treatment‐related AE (not specified) was assessed as serious due to hospitalization of the patient. Nabiximols treatment was withdrawn in 15 (78.9%) of 19 patients with recorded AEs.

**TABLE 3:** Summary of adverse events. Multiple responses were possible

Patients with adverse event, *n*	19
Adverse events, *n*	31
Adverse event by system organ class and preferred term	
Gastrointestinal disorders, *n* (% of total events)	1 (3.2)
Nausea	1
General disorders and administration site conditions, *n* (% of total events)	12 (38.7)
Asthenia	4
Drug ineffective	1
Fatigue	5
Pain	2
Injury, poisoning, and procedural complications, *n* (% of total events)	1 (3.2)
Overdose	1
Investigations, *n* (% of total events)	2 (6.5)
Heart rate increased	1
Respiratory rate increased	1
Nervous system disorders, *n* (% of total events)	13 (41.9)
Gait disturbance	1
Hypertonia	1
Muscle spasticity	1
Muscular weakness	1
Orthostatic hypotension	1
Restlessness	1
Somnolence	2
Vertigo	5
Social circumstances, *n* (% of total events)	1 (3.2)
Immobile	1
Vascular disorders, *n* (% of total events)	1 (3.2)
Hypotension	1

## 4 DISCUSSION

This non‐interventional study prospectively followed Austrian patients with MS spasticity over 3 months to assess the effectiveness and tolerability of nabiximols in the routine outpatient setting. The effectiveness of nabiximols was evaluated primarily with respect to patients’ perceptions of its effect on spasticity‐associated symptoms and impairment of daily activities, and secondarily with respect to patients’ perceptions of its effect on MS spasticity severity and their satisfaction with treatment. The study design was identical to that of the MObility ImproVEment (MOVE)‐2 studies conducted in Germany and EU (mainly Italy) which also tracked evolution in patient‐rated spasticity‐associated symptoms during 3 months’ treatment with nabiximols (Flachenecker et al., 2014b; Vermersch & Trojano, 2016), thus providing a valuable basis for comparison.

The similarity in demographic and clinical characteristics between our cohort and the MOVE‐2 populations (Flachenecker et al., 2014b; Vermersch & Trojano, 2016) suggests that patients were representative of the general population with MS spasticity. Similar to MOVE‐2 Germany (Flachenecker et al., 2014b), at the time of starting treatment with nabiximols, about a third of our patients were not receiving any concomitant antispasticity medications. As nearly all patients (94.5%) had a history of previous antispasticity therapy, mainly baclofen and tizanidine, the absence of underlying treatment at baseline may reflect patients’ general dissatisfaction with conventional antispasticity pharmacotherapy as previously reported (Flachenecker et al., 2014a). Patients’ inability or unwillingness to persist with conventional antispasticity medications due to insufficient benefit or poor tolerability highlights the importance of having alternative options available.

Although the presence of spasticity‐associated symptoms did not change appreciably during nabiximols treatment, patients perceived a reduction in symptom severity. Marked improvement in symptom severity was apparent by 1 month of treatment and further improvement was observed through to 3 months in treatment continuers (72.7% of the effectiveness population). At 3 months, the change from baseline in mean NRS scores for sleep impairment (−47.2%), pain (−40.4%), and MS spasticity (−39.1%) surpassed the 30% threshold for clinically relevant improvement (Farrar et al., 2008), while the 26.4% decrease in the mean fatigue NRS score approached the 30% threshold. Among patients who reported the presence of spasms and/or urinary incontinence at baseline, the numbers of spasms/day and urinary incontinence episodes/week was approximately 70% lower at 3 months than at baseline. MOVE‐2 Germany also reported a rapid response to nabiximols in treatment responders, with the bulk of improvement in mean MS spasticity and sleep disturbance NRS scores apparent by 1 month and maintained at 3 months (Flachenecker et al., 2014b). In MOVE‐2 EU, among patients who completed 3 months’ treatment with nabiximols (65% of the enrolled population), statistically significant decreases from baseline to 3 months were reported for spasms, sleep impairment, night awakenings, fatigue, pain, and urinary incontinence episodes irrespective of whether or not patients had achieved the ≥30% NRS response threshold for MS spasticity (Vermersch & Trojano, 2016).

Despite marked improvement from baseline in the mean sleep impairment NRS score and more than 50% reduction from baseline in the number of patients reporting night awakenings during nabiximols treatment, there was no associated decrease in the number of awakenings/night. Although the reason for this discordance is not clear, it may signal a particularly challenging situation in certain patients with MS spasticity who are prone to multiple sleep interruptions or it may be a study artifact.

No changes were observed during nabiximols treatment with regard to the localization of spasticity‐associated symptoms (which were mainly in the legs) or to the “most disturbing” symptoms as rated by patients (muscle stiffness, restricted mobility). Despite improvement in the frequency or severity of nearly all spasticity‐associated symptoms during nabiximols treatment, there was no associated improvement in patients’ ability to perform daily life activities. This is not entirely unexpected given the relatively advanced stage of MS (51% with secondary progressive MS) and median disability level (EDSS 6) of the patient population at baseline. Moreover, the five‐point categorical scale used to assess daily activity impairment due to spasticity may not be sufficiently sensitive to detect small improvements in spasticity‐associated symptoms that may be meaningful to patients.

Patients’ reported satisfaction with nabiximols was encouraging given their general level of dissatisfaction with previous pharmacotherapy for spasticity‐associated symptoms. Treatment satisfaction is an important element of adherence which, in turn, is a key component of treatment success (Brown & Bussell, 2011). In view of the limited number of options available for patients with MS spasticity who are poorly responsive or intolerant to conventional first‐line medications, maintaining treatment with nabiximols for as long as possible can be a useful strategy to delay or avoid escalation to invasive options.

Nabiximols was well tolerated in this study with no emergence of AEs beyond its known safety profile (eMC, Summary of Product Characteristics, 2022). The stable mean dose of ∼7.3 sprays/day throughout 3 months’ observation suggested that patients maintain the optimal dose achieved during titration, emphasizing the importance of individualized dosing. Although the mean dose was slightly higher than that recorded in other similarly designed observational studies of nabiximols (6−7 sprays/day) (Flachenecker et al., 2014b; Patti et al., 2016; Vermersch & Trojano, 2016), it was well below the recommended maximum of 12 sprays/day (eMC, Summary of Product Characteristics, 2022).

Subgroup analyses of study endpoints revealed no obvious differences according to gender or MS type. These results are not presented due to the small sample sizes.

Supporting our findings is a recent systematic review that evaluated the efficacy of “medical marijuana” in MS and its experimental animal models (Longoria et al., 2022). All clinical studies selected for inclusion in the review involved nabiximols. Overall, there was evidence that add‐on nabiximols improved NRS scores from baseline for spasticity (mean maximal change −2.8 points; nine studies), pain (mean change −3.4 points; five studies), and sleep disturbances (mean change −3.5 points; three studies), and diminished bladder overactivity (three studies) in treatment responders within weeks of starting treatment.

### 4.1 Limitations

Study limitations include those inherent to non‐interventional studies such as the lack of a control group, potential for selection bias, and missing or incomplete data. The planned target of 100 patients could not be reached within a reasonable timeframe due to slow recruitment which was partly due to COVID‐19‐related disruptions. The effectiveness of nabiximols may be overstated since the results do not include data from seven patients who were excluded from the effectiveness population. In addition, mean values reported for assessments conducted after 1 and 3 months of nabiximols treatment derive from treatment continuers at these timepoints, that is, not from treatment discontinuers who can be considered non‐responders. Although it was our intent to capture patients’ own perceptions of symptom severity using patient‐reported assessment tools, we acknowledge the lack of more objective instruments such as the modified Ashworth Scale or Tardieu Scale to measure spasticity. Not least, it is necessary to consider the possible influence of concomitant physiotherapy and inter‐patient variation in rehabilitative techniques on symptom evolution.

## 5 CONCLUSIONS

Within the study limitations, it can be concluded that add‐on nabiximols improved the severity of MS spasticity and a range of spasticity‐associated symptoms during real‐world use in Austria. Nabiximols is an option for patients with MS spasticity who fail first‐line oral antispasticity treatment. Patients with MS spasticity who respond to nabiximols may perceive amelioration of associated symptoms independently of their improvement in spasticity severity.

## AUTHOR CONTRIBUTIONS

Michael Guger, Robert Hatschenberger, and Fritz Leutmezer fulfilled the ICMJE criteria for authorship, which involved initial drafting and critical review of the manuscript during its development and approving the final version for submission.

## Funding information

Almirall GmbH, Vienna, Austria

## CONFLICT OF INTEREST STATEMENT

Michael Guger has received support and honoraria for research, consultation, lectures, and education from Alexion, Almirall, Bayer, Biogen, Celgene, Genzyme, Janssen, MedDay, Merck, Novartis, Ratiopharm, Roche, Sanofi Aventis, and Teva. Robert Hatschenberger has received no financial support within the last 3 years. Fritz Leutmezer has received support and honoraria for research, consultation, lectures, and education from Alexion, Almirall, Bayer, Biogen, Celgene, Genzyme, Janssen, MedDay, Merck, Novartis, Octapharm, Ratiopharm, Roche, Sanofi Aventis, and Teva.

### PEER REVIEW

The peer review history for this article is available at https://publons.com/publon/10.1002/brb3.2947.

## Data Availability

The data that support the findings of this study are available from the corresponding author upon reasonable request.
